# Cytotoxicity and hepatoprotective attributes of methanolic extract of *Rumex vesicarius* L.

**DOI:** 10.1186/s40659-015-0009-8

**Published:** 2015-03-25

**Authors:** Asha Tukappa NK, Ramesh L Londonkar, Hanumantappa B Nayaka, Sanjeev Kumar CB

**Affiliations:** Department of Biotechnology, Gulbarga University, Gulbarga, Karnataka 585106 India

**Keywords:** *Rumex vesicarius* L, HepG2, MDA, SOD, CAT, CCl_4_ induced hepatotocixity

## Abstract

**Background:**

To evaluate the hepatoprotective potential and invitro cytotoxicity studies of whole plant methanol extract of *Rumex vesicarius* L. Methanol extract at a dose of 100 mg/kg bw and 200 mg/kg bw were assessed for its hepatoprotective potential against CCl_4_-induced hepatotoxicity by monitoring activity levels of SGOT (Serum glutamic oxaloacetic transaminase), SGPT (Serum glutamic pyruvic transaminase), ALP (Alkaline phosphatase), TP (Total protein), TB **(**Total bilirubin) and SOD (Superoxide dismutase), CAT (Catalase**)**, MDA (Malondialdehyde). The cytotoxicity of the same extract on HepG2 cell lines were also assessed using MTT assay method at the concentration of 62.5, 125, 250, 500 μg/ml.

**Results:**

Pretreatment of animals with whole plant methanol extracts of *Rumex vesicarius* L. significantly reduced the liver damage and the symptoms of liver injury by restoration of architecture of liver. The biochemical parameters in serum also improved in treated groups compared to the control and standard (silymarin) groups. Histopathological investigation further corroborated these biochemical observations. The cytotoxicity results indicated that the plant extract which were inhibitory to the proliferation of HepG2 cell line with IC50 value of 563.33 ± 0.8 μg/ml were not cytotoxic and appears to be safe.

**Conclusions:**

*Rumex vesicarius* L. whole plant methanol extract exhibit hepatoprotective activity. However the cytotoxicity in HepG2 is inexplicable and warrants further study.

## Background

Liver is the largest glandular organ of the body which not only performs physiological functions but also protects our body from various injurious substance and toxic metabolic byproducts [1]. Inspite of tremendous scientific advancement in the field of hepatology in recent year liver diseases constitute a major problem of worldwide proportions. Jaundice and hepatitis are two major hepatic disorders that account for a high death rate [2]. Liver diseases are mainly caused by toxic chemicals, excess consumption of alcohol, infections and autoimmune disorders [3]. Exposure of the liver to the free radicals derived from some xenobiotic and drugs leads to oxidative stress, which is recognized to be an important factor responsible for liver injury or be involved in the pathogenesis of liver disorder [4,5]. Most of hepatotoxic chemicals damage liver cells mainly by inducing lipid peroxidation and other oxidative damage [3]. In oxidation process, highly reactive and harmful chain reactions of oxygen species are generated, causing damage to living organisms. The oxygen centered free radicals and other reactive oxygen species (ROS), which are continuously produced, has resulted in cell death or tissue damage. This oxidation damage caused by free radical is related to pathogenesis of many chronic degenerative diseases like cancer, diabetes, neurodegenerative diseases, atherosclerosis, cirrhosis, malaria and Acquired immune deficiency syndrome (AIDS) [6]. Hence, antioxidants may play a role in inhibiting the liver injury induced during cell damage and attribute to the hepatoprotection [7].

Antioxidant is a molecule which terminates the chain reaction by removing free radical intermediate. However plants and animals maintain complex system of multiple types of antioxidants. And also the natural plant based antioxidants have played an important role in the maintence of human health for past three decades [8]. There are a number of medicinal preparations in ayurveda that are recommended for the treatment of liver disorders [9]. Inspite of tremendous advances in modern medicine there are no effective and reliable drugs available that can stimulate liver function, offer protection to the tissue damage and help to regenerate cells [10].

Food rich in natural antioxidants have been proposed as a tool to prevent and cure liver damage [11]. The plants belonging to Polygonaceae family are generally a rich source of substances of phytochemical interest. Number of plants from this family is used in traditional system of medicine [12]. From the large list of various plants of Polygonaceae family we have selected *Rumex vesicarius* Linn. The review of literature showed that there are so many important phytoconstituents present in the various parts of the *Rumex vesicarius* and they are responsible for some important biological activities like antimicrobial activity, antitumor activity, wound healing and anti-inflammatory [13].

*Rumex vesicarius* Linn. is a branched succulent herb which belongs to the family Polygonaceae and is distributed in India. The whole plant is medicinally important and cures several diseases thus traditionally used as asperients, diuretic and cooling agent. The plant juice is useful in curing stomach heat, toothache, checks nausea and promotes appetite. Fruits are asperients and diuretic, eaten fresh against Jaundice, hepatic conditions, constipation and indigestion, rosted seeds are prescribed in dysentery [14,15].

Keeping these folkloric claims and reports in view, the present study is attempted to evaluate the possible hepatoprotective and antioxidant effect of methanolic extract of *Rumex vesicarius* Linn. in CCl_4_ induced hepatotoxicity in rats and cytotoxic effect on HepG2 cell lines.

## Result and discussion

The present study is attempted to demonstrate the role of hepatoprotective activity of whole plant methanol extract of *Rumex vesicarius* L. in CCl_4_ induced hepatotoxicity at different dose (100 mg and 200 mg/kg bw) and Cytotoxic activity on HepG2 cell lines.

### Hepatoprotective activity

#### Body and organ weight

Organ weight and body weight decrease is an indication of organ injury. Organ weight changes have long been accepted as a sensitive indicator of chemically induced changes to organs. In toxicological experiments, comparison of organ weights between treated and untreated groups of animals have conventionally been used to evaluate the toxic effect of the test drug [16,17]. From the data presented in Table 1, it could be noticed that the body weight and the organ (liver and kidney) weight differed significantly between the CCl_4_ treated group and the normal control group. However, pretreatment of *Rumex vesicarius* L. have reduced the effect of CCl_4_ on rat’s body weight and organ weight.

**Table 1:** **Effect of methanolic extract of** ***Rumex vesicarius*** **L. on body weight and organ weight compared to silymarin in liver damaged rats**

**Groups**	**Dosing**	**Body weight**		**Relative organ weight**		
		**Before**	**After**	**Liver**	**Kidney**	
					**Right side**	**Left side**
Group I	1%Tween 80 5 ml/kg bw.p.o	112 ± 6.23	116 ± 8.164	4.656 ± 0.322	0.442 ± 0.015	0.449 ± 0.023
Group II	CCl4 1.5 ml/kg bw.i.p	119 ± 8.34	120 ± 10.80	4.163 ± 0.22	0.425 ± 0.013	0.405 ± 0.01
Group III	Silymarin 50 mg/kgbw.p.o	113 ± 10.12	116 ± 10.80	4.582 ± 0.873	0.45 ± 0.038	0.452 ± 0.029
Group IV	100 mg/kg bw.p.o	104 ± 8.65	105 ± 8.164	4.22 ± 0.728	0.475 ± 0.040	0.413 ± 0.028
Group V	200 mg/kgbw.p.o	108 ± 10.21	111 ± 10.80	4.501 ± 0.124	0.450 ± 0.016	0.447 ± 0.009

Each value represents the mean of 6 rats ± S.E. significant at *P >* 0.05, as compared with the corresponding control group.

### Food and water intake

**Figure 1:**
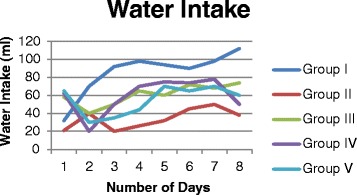
**Effect of methanol extract of** ***Rumex vesicarius*** **L on water consumption in CCl4 toxicity induced rats.** Group I: (control), Group II: (CCl4 treated), Group III: (pretreatment with silymarin), Group IV: (pretreatment of extract at low dose of 100 mg/kgbw), Group V: (pretreatment of extract at low dose of 200 mg/kgbw).

**Figure 2:**
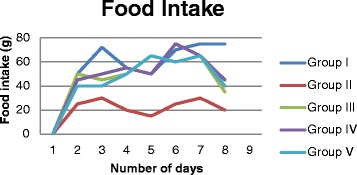
**Effect of methanol extract of** ***Rumex vesicarius*** **L on food consumption in CCl**_**4**_ **toxicity induced rats.** Group I: (control), Group II: (CCl4 treated), Group III: (pretreatment with silymarin), Group IV: (pretreatment of extract at low dose of 100 mg/kgbw), Group V: (pretreatment of extract at low dose of 200 mg/kgbw).

The amount of water and food intake with the animals treated with vehicle and silymarin were normal and there was no significant difference found between these groups over the period of treatment (Figures 1 and 2). The animals administrated with CCl_4_ (toxic group), failed to eat and drink normally when compared to vehicle and silymarin treated groups. However pretreatment of methanol extract of *Rumex vesicarius* L. reduced the effect of CCl_4_ on food and water consumption. These results are in agreement with the work carried out by Nirwane [18]. They investigated possible hepatoprotective effect of *Piper nigrum* using CCl_4_ induced hepatotoxicity and reported that pretreatment of methanol extract reduced the effect of CCl_4_ on food and water consumption.

### Hepatotoxicity assessment

Liver is a key organ regulating homeostasis in body. It also plays a major role in detoxification and excretion of many endogenous and exogenous compounds and injury of its function may lead to several implications on human health [19]. It has been reported that hepatotoxic chemicals, in general, release free radicals (ROS, RNS), which act on hepatic cells, especially in the liver parenchyma cells [20]. The hepatic parenchyma cells are the production site for various enzymes, bile salts and bile pigments etc. Damage caused by hepatotoxin_,_ increases the level of liver enzymes. Liver damage induced by CCl_4_ is commonly used model for the screening of hepatoprotective drugs. Thus in the present study CCl_4_, a hepatotoxin is used to induce acute liver damage. Alkaline phosphate is a membrane bound glycoprotein enzyme related to the functioning of hepatocytes and increase in its activity is due to the increased synthesis in presence of biliary pressure. However protection of hepatocytes and stabilization of plasma membrane against the damage caused by hepatotoxin is indicated by decrease in the levels of transaminase and also reduction of alkaline phosphatase levels with concurrent depletion of raised bilirubin levels suggests the stability of the biliary function during injury with CCl_4_ [21].

The rise in serum levels of SGOT, SGPT, ALP, TB and TP, has been attributed to the damaged structural integrity of the liver, because they are cytoplasmic in location and released into circulation after cellular damages [22]. When rats were treated with carbon tetrachloride it induces hepatotoxicity by metabolic activation, therefore, it selectively causes toxicity in liver cells maintaining semi-normal metabolic function. Carbon tetrachloride is metabolically activated by the cytochrome P-450 dependent mixed oxidase in the endoplasmic reticulum to form trichloromethyl free radical (CCl_3_) which combined with cellular lipids and proteins in the presence of oxygen to induce lipid per-oxidation [23-25]. These result in change of structure of the endoplasmic reticulum and other membrane, loss of metabolic enzyme activation, reduction of protein synthesis and loss of glucose-6-phosphatase activation, leading to liver injury [26].

The biochemical results of hepatoprotective activities of methanol extract of *Rumex vesicarius* L. at dose of 100 mg and 200 mg/kg bw on rats intoxicated with carbon tetrachloride were illustrated in the Table. 2. Results showed that CCl_4_ induced a rise of the serum enzymes which are well known markers of hepatic injury. There is an increase in levels of SGPT (Serum glutamic pyruvic transaminase), SGOT (serum glutamic oxaloacetic transaminase), ALP (Alkaline phosphatase) with the value of 66.09 ± 2.469, 275.0 ± 2.987, 492.5 ± 4.891 U/L respectively and TB **(**Total bilirubin) with 1.41 ± 0.08 mg/dl with a significant decrease in TP (Total protein) with 4.93 ± 0.13 mg/dl in CCl_4_ intoxicated rats as compared to the normal control group with the value of 43.15 ± 3.832 U/L, 119.26 ± 4.650 U/L, 440.7 ± 7.07 U/L, 0.70 ± 0.05 mg/dl, 6.39 ± 0.35mg/dl for SGPT, SGOT, ALP, TP and TB respectively.

**Table 2:** **Effect of methanolic extract of** ***Rumex vesicarius*** **L on plasma enzymes (SGOT, SGPT, ALP), Cholesterol, total bilirubin (T.B) and total protein (T.P) compared to silymarin in liver damaged rats**

**Groups**	**Dosing**	**SGOT (U/L)**	**SGPT (U/L)**	**ALP (U/L)**	**T.P (mg/dL)**	**T.B (mg/dL)**
Group I	1%Tween 80 5 ml/kg bw.p.o	119.26 ± 4.650	43.15 ± 3.832	440.7 ± 7.07	6.39 ± 0.35	0.70 ± 0.05
Group II	CCl4 1.5 ml/kg bw.i.p	275.0 ± 2.987	66.09 ± 2.469	492.5 ± 4.891	4.93 ± 0.13	1.41 ± 0.08
Group III	Silymarin 50 mg/kgbw.p.o	132.0 ± 0.653	45.96 ± 2.029	275.3 ± 3.150	5.52 ± 0.36	0.86 ± 0.04
Group IV	100 mg/kg bw.p.o	210.5 ± 0.697	62.52 ± 2.063	214.3 ± 2.843	5.03 ± 0.28	0.91 ± 0.03
Group V	200 mg/kgbw.p.o	177.3 ± 2.970	53.03 ± 2.95	308.7 ± 2.465	5.79 ± 0.23	0.98 ± 0.04

Each value represents the mean of 6 rats ± S.E. significant at *P >* 0.0001; as compared with the corresponding control group.

SGOT- serum glutamic oxaloacetic transaminase, SGPT -serum glutamic pyruvic transaminase, ALP**-** Alkaline phosphatase,

TP- Total protein TB **-**Total bilirubin.

However, pretreatment of *Rumex vesicarius* L extract (100 mg/kg bw and 200 mg/kg bw) and silymarin significantly preserved biochemical changes during CCl_4_ intoxification and confirmed their potential hepatoprotection activity to accelerate regeneration of parenchyma cells. There is fall down in the level of SGPT, SGOT, ALP and TB in the rats treated with 100 mg/kgbw, 200 mg/kgbw of *Rumex vesicarius* extract and silymarin with the values of 62.52 ± 2.063 U/L, 210.5 ± 0.697 U/L, 214.3 ± 2.843 U/L. 0.91 ± 0.03 mg/dl.where as significant increase in TP with value of 5.03 ± 0.28 mg/dl for 100 mg/kg bw of *Rumex vesicarius* and 53.03 ± 2.95 U/L, 177.3 ± 2.970 U/L, 308.7 ± 2.465 U/L, 0.98 ± 0.04 mg/dl, 5.79 ± 0.23 mg/dl of SGPT, SGOT, ALP , TB, TP respectively for high dose (200 mg/kgbw) of *Rumex vesicarius* L. which are in coordination with the results obtained for silymarin with the values of 45.96 ± 2.029 U/L, 132.0 ± 0.653 U/L, 275.3 ± 3.150 U/L for SGPT, SGOT, ALP respectively and 0.86 ± 0.04 mg/dl, 5.52 ± 0.36 mg/dl for TB and TP respectively.

The elevated serum protein level suggested the stabilization of endoplasmic reticulum leading to an increase in protein synthesis that enhanced hepatocyte regeneration [27]. On the other hand reduction in the ALP level with concurrent depletion of raised bilirubin level suggested the stability of the biliary function.

### Antioxidant enzyme activity assay

The body has an effective antioxidant system against free radicals and ROS induced damage in which the endogenous enzymatic and non enzymatic antioxidants such as SOD, CAT and MDA play an important role [28,29]. SOD and CAT antioxidant enzymes constitute a mutually supportive team of defense against ROS. The elevated level of MDA indicates excessive formation of free radicals and activation of lipid peroxidation system resulting in hepatic damage. MDA produced as byproduct by lipid peroxidation that occurs in hydrophobic core of biomembrane.

In the present study results in Table 3 proved that intoxicaton with CCl_4_ caused a disturbance in the antioxidant defense systems and oxidative stress as evident from a marked decreased in the antioxidant enzyme activities SOD and CAT along with increase in the MDA. The SOD and CAT values are found to be 2.23 ± 0.15 U/mg Hb and 2.27 ± 0.10 U/mg Hb compared to control group with 3.48 ± 0.16 U/mg Hb 2.78 ± 0.09 U/mg Hb respectively. The elevated level of MDA in CCl_4_ treated group with 24.2 ± 0.76 nmol/mg compared to control group with 12.4 ± 0.48 nmol/mg, indicates excessive formation of free radicals and activation of lipid peroxidation system resulting in hepatic damage.

**Table 3:** **Effect of methanolic extract of** ***Rumex vesicarius*** **L on SOD, CAT, MDA compared to silymarin in liver damaged rats**

**Groups**	**Dosing**	**SOD (U/mg Hb)**	**CAT (U/mg Hb)**	**MDA (nmol/mg)**
Group I	1%Tween 80			
Control	5 ml/kg bw.p.o	3.48 ± 0.16	2.78 ± 0.09	12.4 ± 0.48
Group II	CCl4	2.23 ± 0.15	2.27 ± 0.10	24.2 ± 0.76
Toxic	1.5 ml/kg bw.i.p			
Group III	Silymarin	4.52 ± 0.24	3.27 ± 0.11	15.3 ± 0.67
Positive control	50 mg/kgbw.p.o			
Group IV	100 mg/kg bw.p.o	3.63 ± 0.15	2.87 ± 0.03	17.1 ± 0.53
Low dose				
Group V	200 mg/kgbw.p.o	4.18 ± 0.24	3.35 ± 0.14	14.6 ± 0.54
High dose				

Each value represents the mean of 6 rats ± S.E. significant at *P <* 0.0001; as compared with the corresponding control group.

SOD: superoxide dismutase; CAT: catalase; MDA = malondialdehyde.

The pretreatment of *Rumex vesicarius* L.extract at the dose of 100 mg/kgbw, 200 mg/kgbw and silymarin at the dose of 50 mg/kgbw significantly increased SOD with 3.63 ± 0.15 U/mg Hb, 4.18 ± 0.24 U/mg Hb, 4.52 ± 0.24 U/mg Hb and CAT with 2.87 ± 0.03 U/mg Hb, 3.35 ± 0.14 U/mg Hb, 3.27 ± 0.11 U/mg Hb respectively. There is significant decline in the concentration of MDA in the rats pretreated with *Rumex vesicarius* L. extract of 100 mg/kgbw, 200 mg/kg bw and silymarin with 17.1 ± 0.53 nmol/mg, 14.6 ± 0.54 nmol/mg, 15.3 ± 0.67 nmol/mg respectively, indicates antilipid peroxidation effect of *Rumex vesicarius* L. (Table 3), also the pretreatment of *Rumex vesicarius* L. effectively blocked CCl_4_ induced abnormal changes in the liver and activity of SOD and CAT in rats reveals that *Rumex vesicarius* L. have a potent antioxidant property towards chemical induced hepatic injury.

### Histopathological studies

**Figure 3:**
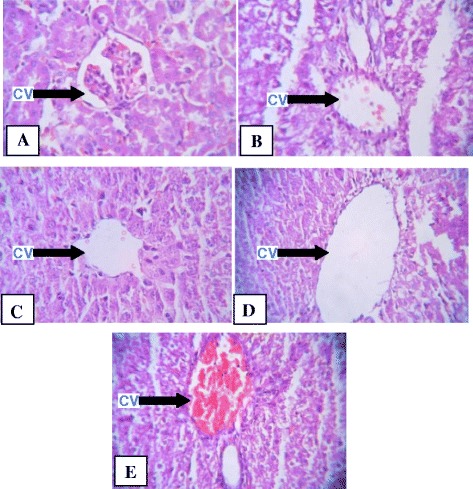
**Effect of methanolic extract of** ***Rumex vesicarius*** **L on acute liver injury induced by CCl**_**4**_ **.** **A**: (control): liver section with normal structure and architecture. **B**: (CCl4 treated): showing extensive area of necrosis, profound inflammation and congestion. **C**: (pretreatment with silymarin): reduced inflammation and degenerative changes. **D**: (pretreatment of extract at low dose of 100 mg/kgbw): reduced inflammation, degenerative changes. **E**: (pretreatment of extract at low dose of 200mg/kgbw): reduced inflammation, degenerative changes. cv: central vein

The results of histopathological studies provided supportive evidence for biochemical analysis. Histology of liver section of normal control animal exhibit normal hepatic cells each with well defined cytoplasm, prominent nucleus and nucleolus and well brought central vein (Figure 3A). The CCl_4_ intoxicated group animals showed total loss of hepatic architecture with centrilobular hepatic necrosis, fatty changes vacuolization and congestion of sinusoids, kupffer cell hyperplasia, crowding of central vein and apoptosis (Figure 3B). Pretreatment with *Rumex vesicarius* L. at the dose of 100 mg/kgbw and 200 mg/kgbw and silymarin showed protecting activity (Figure 3C-E). The methanol extract of *Rumex vesicarius* L. at low dose of 100 mg/kg bw. showed moderate or weak hepatoprotective activity for CCl_4_ injury. However the methanol extract at the high dose of 200 mg/kg bw and silymarin had shown a potential hepatoprotective activity and reduced the degenerative changes in liver. The result thus obtained is in the agreement with the work carried out by Sahreen [26]. They reported the hepatoprotective activity of methanol extract of *Carissa opaca* leaves on CCl_4_ induced damage in rat.

### Cytotoxicity

Plants contain almost unlimited capacity to generate compounds that fascinates researchers in the quest for new and novel chemotherapeutics [30]. The persistency search for new compounds in medicinal plant and traditional food is a realistic and promising strategy for prevention of diseases [31]. Therefore in this study *Rumex vesicarius* L. was evaluated for its Cytotoxicity on HepG2 (human hepatocarcinoma) cells using MTT (3-(4, 5-dimethylythiazol-2-yl)-2, 5-diphenyl-2H-tetrazolium hydrobromide) assay. The ability of the cells to survive a toxic insult has been the basis of most Cytotoxicity assays.

In the present study cytotoxicity of whole plant methanol extract of *Rumex vesicarius* L. at different concentration (62.5 μg/ml, 125 μg/ml, 250 μg/ml, 500 μg/ml, and 1000 μg/ml) were presented in Table 4. The % cytotoxicity of methanol extract of *Rumex vesicarius* L. was found to be dose dependent and increases with increased concentrations. HepG2 experienced significant decrease in viability at low concentration of methanol extract of *Rumex vesicarius* L. with eventual decline at highest concentration tested. The % inhibition of cell proliferation at 62.5 μg/ml, 125 μg/ml, 250 μg/ml, 500 μg/ml, 1000 μg/ml was found to be 21.57 ± 0.7, 32.68 ± 1.0, 41.69 ± 0.6, 47.92 ± 0.9, 70.21 ± 0.6 respectively, with an IC50 value of 563.88 ± 0.8 μg/ml.

**Table 4:** **Cytotoxic properties of methanolic extract of** ***Rumex vesicarius*** **L. against HepG2 cell line**

**Sl. no**	**Test conc.(μg/ml)**	**% Cytotoxicity**	**CTC**_**50**_ **(μg/ml)**
1	1000	70.21 ± 0.6	
2	500	47.92 ± 0.9	
3	250	41.69 ± 0.6	563.33 ± 0.8
4	125	32.68 ± 1.0	
5	62.5	21.57 ± 0.7	

A study carried out by Patel evaluated that methanolic extract of *S. nigrum* fruits has potential activity on HeLa cell and lesser effect on Vero cell. So, the drug has considerable anticancer activity on cervical cancer [32]. Wang *et al*. showed antitumor effects of methanolic extract of *S. nigrum* fruits on U266 cells [33]. During Wang *et al*.’s search for new anti-tumor agents, the ethanolic extract of fruits of *C. pepo* was observed to exhibit a significant dose-dependent inhibitory effect against HeLa cell growth [34].

Magdalena [35] investigated in vitro screening of cytotoxic activities of ethanol extract from roots, leaves and fruits of six *Rumex* species: *R. acetosa* L., *R. acetosella* L., *R. confertus* Willd., *R. crispus* L., *R. hydrolapathum* Huds. and *R. obtusifolius* L. on leukemic 1301 and EOL-1 cell lines. There is however sources confirming strong cytotoxic properties of the compounds that have been isolated from *Rumex acetosa* against tumor cell lines. Chrysophanol, Physcion, emodin and emodin-8-O-β-D-glucopiranoside were isolated from methanol extract of *Rumex acetosa*. They showed high cytotoxicity activity against 5 human tumor cell lines A549 lung cancer, overian Sk-OV-3, Central nerous system XF 498, Intestines HCT-15 and MEC-2 melanoma.

When examining cytotoxicity properties of Dock species, two herbal preparation Essiac® and Flor Essenc® which include herb of *Rumex acetosella*. It has been also proved that those herbal tonic induced proliferation of T47D, MDA-MB-231 and MDA-MB-436 breast cancer cell lines [36-38]. Recent studies have confirmed the properties of many cytotoxic compounds from roots of *Rumex crispus*, responsible for killing cells lines DLD-1 (human colon adenocarcinoma cell line) [39].

In the present study the methanol extract showed invitro cytotoxicity and invivo hepatoprotective potential. As per our earlier findings, the methanol extract consist of many secondary metabolites like phenols, flavonoids, alkaloids, glycosides. The cytotoxicity and hepatoprotective attributes may be due to these different secondary metabolites which may have a different pharmacological activity.

## Conclusion

The present study demonstrated the protective activity of *Rumex vesicarius* L. against CCl_4_ induced hepatotoxicity might be due to its antioxidant potential. The hepatoprotective role of *Rumex vesicarius* L. extract was found to be comparable with silymarin which might be due to the presence of flavonoid providing a new way to develop potential hepatoprotective drugs. The *Rumex vesicarius* L. allows us to conclude that extracts is good candidates for further studies of activity-monitored fractionation to identify their active components. Cytotoxicity study of methanol extract in HepG2 is however inexplicable and warrants further study.

## Methods

### Plant material

The plant *Rumex vesicarius*. Linn was collected from village Khusnoor, Gulbarga district, Karnataka in the month of July- August and authenticated by Department of Botany, Gulbarga University, Gulbarga. The Voucher specimen was submitted at Department of Botany with the reference No. HGUG-5012.

### Preparation of extract

The powdered crude drug of *Rumex vesicarius* L. was successively extracted with petroleum ether, chloroform and methanol by soxhlet method. The extract was filtered and concentrated to dryness at room temperature. Our earlier qualitative chemical investigation of petroleum ether, chloroform and methanol extract reveals, the presences of secondary metabolites in methanol extract are high [40]. Thus methanol extract was selected for further study.

### Hepatoprotective activity

#### Drugs and chemicals

Silymarin was purchased from Microlabs Limited, Goa. All other chemicals used in this study were of analytical grade.

### Experimental animals

Male albino rats of Wistar strain weighing between 100 and 130 g (8 weeks old) were purchased from the Animal House Colony of the Luqman Pharmacy College, Gulbarga. The animals were housed in polypropylene cages and maintained at 25 ± 2°C under 14/10 h dark and light cycle. They were allowed free access to standard pellet diet and water *ad libitum*. The animals were acclimatized for one week under laboratory conditions. Ethical clearance for handling the animals was obtained from the ethical committee constituted for the purpose (CPCSEA Reg. No. 34800/2001).

### Animal dose

Based on our earlier studies of acute and short term toxicities the appropriate dose for preclinical study was calculated and found to be 100 mg/kg body weight (low dose) and 200 mg/kg body weight (high dose) (communicated data).

### Experimental design

Rats were divided into 5 groups, each with 6 animals:

- Group I Control (vehicle): Received 1% v/v Tween-80 in distilled water (5 ml/kg body weight, p.o.) single daily dose for 6 consecutive days.
- Group II (CCl_4_ induced): Received 1% v/v Tween-80 in distilled water (5 ml/kg body weight, p.o.) single daily dose for 6 consecutive days.
- Group III (standard): Received standard drug Silymarin (100 mg/kg body weight p.o.) single daily dose for 6 consecutive days.
- Group IV (Low dose): Received methanol extract of *Rumex vesicarius* L. (100 mg/kg body weight, p.o.) single daily dose for 6 consecutive days.
- Group V (High dose): Received methanol extract *Rumex vesicarius* L. (200 mg/kg body weight, p.o.) single daily dose for 6 consecutive days.

Groups from II to V have received CCl_4_ in olive oil (1:1 v/v, 1.5 ml/kg body weight i.p.) single dose on the 7th day. The quantity of food and water consumed was recorded for each group of animals during the course of the experiment. The body a weight of each rat was recorded on the 1st day and on 8th day, mean body weights were calculated. Liver weight and kidney weight were also determined to know the effect of drug on normal morphology and physiology of rats. Organ index was calculated according to the formula: (liver or kidney weight/body weight) × 100%.

### Sample collection

All rats were sacrificed by cervical decapitation separately after 24 hrs of the last treatment on 8th day. Blood samples of each group were collected into dry sterilized tubes and centrifuged at 3000 r/min. for 10 min to obtain clear serum. Liver and Kidney were quickly excised, rinsed in cold saline, blotted, and weighed. A part of liver was freshly used for assay of malondialdehyde (MDA) and another part is used for histopathological examination.

### Hepatotoxicity assessment

The hepatic enzymes SGOT and SGPT were used as the biochemical indicators for the acute liver injury. The serum SGOT, SGPT, ALP, TB and TP activities were determined by using a commercial diagnostic kit Biocompare, BioVision, Thermo scientific and Biooscientific Max discovery.

### Antioxidant enzyme activity assay

#### Preparation of erythrocyte lysate

The packed cells, after separation of serum, were washed with a physiological saline 3 times and lysed with hypotonic phosphate buffer, pH 7.4. The hemolysate was separated by centrifuging at 2500 r/min for 15 min at 4°C and used for assay of erythrocyte superoxide dismutase (SOD) and catalase (CAT) activities.

### Superoxide dismutase (SOD)

This method is well described by McCord and Fridovich and can be applied for determination of antioxidant activity of a sample. It is estimated in the erythrocyte lysate prepared from the 5% RBC suspension. To 50 lL of the lysate, 75 mM of Tris–HCl buffer (pH 8.2), 30 mM EDTA and 2 mM of pyrogallol are added. An increase in absorbance is recorded at 420 nm for 3 min by spectrophotometer. One unit of enzyme activity is 50% inhibition of the rate of autooxidation of pyrogallol as determined by change in absorbance/min at 420 nm. The activity of SOD is expressed as units/mg protein [41].

### Catalase (CAT)

Catalase activity can be determined in erythrocyte lysate using Aebi’s method [42]. Fifty micro liter of the lysate is added to a cuvette containing 2 mL of phosphate buffer (pH 7.0) and 1 mL of 30 mM H_2_O_2_. Catalase activity is measured at 240 nm for 1 min using spectrophotometer. The molar extinction coefficient of H_2_O_2_, 43.6 M cm_1 was used to determine the catalase activity. One unit of activity is equal to1 mmol of H_2_O_2_ degraded per minute and is expressed as units per milligram of protein.

### Lipid Peroxidation (LPO) assay

The liver tissues were homogenized in 10% ice-cold phosphate buffered saline (0.1 M, pH 7.4, w/v) and centrifuged at 2000 r/min for 30 min; the supernatant was used for the assay of MDA.

LPO is an autocatalytic process, which is a common consequence of cell death. This process may cause peroxidative tissue damage in inflammation, cancer and toxicity of xenobiotics and aging. Malondialdehyde (MDA) is one of the end products in the lipid peroxidation process. Malondialdehyde (MDA) is formed during oxidative degeneration as a product of free oxygen radicals, which is accepted as an indicator of lipid peroxidation. This method described by Ohkawa is as follows: The tissues are homogenized in 0.1 M buffer pH 7.4 with a Teflonglass homogenizer. LPO in this homogenate is determined by measuring the amounts of malondialdehyde (MDA) produced primarily. To tissue homogenate (0.2 mL), 0.2 mL of 8.1% sodium dodecyl sulfate (SDS), 1.5 mL of 20% acetic acid and 1.5 mL of 8% TBA are added. The volume of the mixture is made up to 4 mL with distilled water and then heated at 95°C on a water bath for 60 min using glass balls as condenser. After incubation the tubes were cooled to room temperature and final volume was made to 5 mL in each tube. Five mL of butanol: pyridine (15:1) mixture is added and the contents are vortexed thoroughly for 2 min. After centrifugation at 3000 rpm for 10 min, the upper organic layer is taken and its OD is taken at 532 nm against an appropriate blank without the sample. The levels of lipid peroxides can be expressed as moles of thiobarbituric acid reactive substances (TBARS)/mg protein using an extinction coefficient of 1.56 · 10 5ML cm_1 [43].

### Histopathological studies

The liver tissues were removed from the animals and immediately fixed in 10% formalin. Subsequent processing included dehydrating in increasing ethanol solutions (50–100%), clearing in xylene and embedding in paraffin. Sections (4–5 μm) were prepared and then stained with Haematoxylin/Eosin dye for photo microscopic observation.

### Cytotoxicity

#### Chemicals

3-(4,5–dimethyl thiazol–2–yl)–5–diphenyl tetrazolium bromide (MTT), Fetal Bovine serum (FBS), Phosphate Buffered Saline (PBS), Dulbecco’s Modified Eagle’s Medium (DMEM) and Trypsin were obtained from Sigma Aldrich Co, St Louis, USA. EDTA, Glucose and antibiotics from Hi-Media Laboratories Ltd., Mumbai. Dimethyl Sulfoxide (DMSO) and Propanol from E.Merck Ltd., Mumbai, India.

### Cell lines and culture medium

HepG2 (human hepatocarcinoma) cell lines were procured from National Centre for Cell Sciences (NCCS), Pune, India. Stock cells were cultured in DMEM supplemented with 10% inactivated Fetal Bovine Serum (FBS), penicillin (100 IU/ml), streptomycin (100 μg/ml) and amphotericin B (5 μg/ml) in an humidified atmosphere of 5% CO_2_ at 37°C until confluent. The cells were dissociated with TPVG solution (0.2% trypsin, 0.02% EDTA, 0.05% glucose in PBS). The stock cultures were grown in 25 cm^2^ culture flasks and all experiments were carried out in 96 microtitre plates (Tarsons India Pvt. Ltd., Kolkata, India).

### Preparation of test solution

For Cytotoxicity studies, weighed extract was dissolved in distilled DMSO and volume was made up with DMEM supplemented with 2% inactivated FBS to obtain a stock solution of 1 mg/ml concentration and sterilized by filtration. Serial two fold dilutions were prepared from the stock to carry out cytotoxic studies.

### MTT assay

The monolayer cell culture was trypsinized and the cell count was adjusted to 1.0 × 10^5^ cells/ml using DMEM containing 10% FBS. To each well of the 96 well microtitre plate, 0.1 ml of the diluted cell suspension (approximately 10,000 cells) was added. After 24 h, when a partial monolayer was formed, the supernatant was flicked off, washed the monolayer once with medium and 100 μl of different test concentrations of test drugs were added on to the partial monolayer in microtitre plates. The plates were then incubated at 37°C for 3 days in 5% CO_2_ atmosphere, and microscopic examination was carried out and observations were noted every 24 h interval. After 72 h, the drug solutions in the wells were discarded and 50 μl of MTT in PBS was added to each well. The plates were gently shaken and incubated for 3 h at 37°C in 5% CO_2_ atmosphere. The supernatant was removed and 100 μl of propanol was added and the plates were gently shaken to solubilize the formed formazan. The absorbance was measured using a microplate reader at a wavelength of 540 nm. The percentage growth inhibition was calculated using the following formula and concentration of test drug needed to inhibit cell growth by 50% (CTC_50_) values is generated from the dose–response curves for each cell line [44].

% Growth inhibition = 100- [Mean OD of individual test group/Mean OD of control group × 100].

### Statistical analysis

Statistical analysis was performed using Graph pad (version 6.04).The data were expressed as means ± SD, and significant differences were determined by One-way analysis of Variance (ANOVA) followed with Duncan’s multiple range tests. A *p*-value of less than 0.01 was considered statistically significant.

## Abbreviations

p.o: Per os
i.p: Intraperitoneal
bw: Body weight
v/v: Volume/volume
w/v: Weight/volume
